# A Study of Molecular Dynamic Simulation and Experimental Performance of the Eucommia Ulmoides Gum-Modified Asphalt

**DOI:** 10.3390/ma16165700

**Published:** 2023-08-19

**Authors:** Simeng Yan, Naisheng Guo, Zhaoyang Chu, Xin Jin, Chenze Fang, Sitong Yan

**Affiliations:** 1 College of Transportation Engineering, Dalian Maritime University, Dalian 116026, China; 0120180086@dlmu.edu.cn (S.Y.); chzy@dlmu.edu.cn (Z.C.); fangchenze@126.com (C.F.); 2 College of Communication, Tonghua Normal University, Tonghua 134002, China; yansitong1128@126.com; 3 School of Transportation Engineering, Shenyang Jianzhu University, Shenyang 110168, China; jinxinzzz@126.com

**Keywords:** asphalt, EUG, gutta-percha-modified asphalt, molecular dynamic simulation, compatibility

## Abstract

In recent years, eucommia ulmoides gum (EUG), also known as gutta-percha, has been extensively researched. Molecular dynamic simulations and experiments were used together to look at how well gutta-percha and asphalt work together and how gutta-percha-modified asphalt works. To investigate the gutta-percha and asphalt blending systems, the molecular models of asphalt and various dosages of gutta-percha-modified asphalt were set up using Materials Studio (MS), and the solubility parameters, intermolecular interaction energy, diffusion coefficient, and mechanical properties (including elastic modulus, bulk modulus, and shear modulus) of each system were calculated using molecular dynamic simulations at various temperatures. The findings indicate that EUG and asphalt are compatible, and sulfurized eucommia ulmoides gum (SEUG) and asphalt are more compatible than EUG. However, SEUG-modified asphalt has better mechanical properties than EUG, and the best preparation conditions are 10 wt% doping and 1 h of 180 °C shearing. Primarily, physical modifications are required for gutta-percha-modified asphalt.

## 1. Introduction

At present, global demand for bitumen has been increasing over the last few decades. In 2010, the global bitumen capacity was 164 million tons and production was 119 million tons; by 2021, the global bitumen industry will have a capacity of 219 million tons and a production of 139 million tons, an increase of 33.53% in capacity and 16.8% in production over eleven years. The global bitumen industry is a major contributor to infrastructure investment in roads, airports, and ports [1]. Since 1980, the modification of asphalt to reduce the temperature sensitivity of asphalt pavement and reduce problems, such as rutting and cracking of pavement, has become a very important technological tool [2]. According to their effectiveness, modifiers can be divided into four main types: adhesive, plasticizing, structural, and composite [3,4]. Among them, polymeric materials, as one of the composite modifiers, can significantly improve the permanent deformation resistance, temperature shrinkage cracking resistance, and fatigue cracking resistance of pavement compared with ordinary asphalt, and, therefore, are widely used in road construction [2,5,6].

Polymer modifiers such as polyethylene (PE), atactic polypropylene (APP), styrene-butadiene latex (SBR), polystyrene–butadiene–styrene block copolymers (SBS), and epoxy resins are mostly by-products of petroleum cracking [6,7,8]. Among them, SBS has become the most widely used modifier in road construction both domestically and abroad due to its excellent high- and low-temperature resistance and anti-aging properties [9]. However, there are also shortcomings such as poor compatibility between SBS and asphalt, poor storage stability of SBS-modified asphalt, insufficient performance in complex environments, such as high temperatures, strong ultraviolet rays, heavy loads, etc., and the price of SBS is expensive and not environmentally friendly [7,10]. Especially in recent years, building an ecological civilization and creating a better living environment has become the consensus of our government and the nation. Since 2020, China has introduced a series of policies to build and promote sustainable green transport [11,12,13]. Sooner or later, traditional SBS modifiers will be replaced by high-performance, high-value-added, resource-saving, and environmentally friendly green materials [14]. And dulcimer gum is a promising green eco-resource [15,16].

The natural polymer material EUG is mainly produced from the leaves, bark, and seeds of eucommia, a unique forest tree species in China. EUG is composed of trans-polyisoprene and, although it is isomeric to natural rubber (NR), its performance varies widely. Unlike NR elastomeric grades, EUG has good biocompatibility, insulation, acid and alkali resistance, hydrophobicity, mechanical properties, and rubber–plastic duality. Therefore, thermoplastic, thermoelastic, and rubber-like materials can be developed by modulating the degree of cross-linking of EUG, and new composites with strong functionality can be developed by blending rubber, plastics, nanomaterials, and other materials [17,18,19,20,21,22,23,24]. In recent years, EUG has attracted much attention as a green and promising new bio-based material [25,26]. Based on EUG, researchers have developed green tires, shock- and sound-absorbing materials, shape-memory materials, medical materials, and biodegradable composites [27,28,29,30,31]. In addition, researchers have investigated the application of EUG as a modifier material in the road sector [26].

Li et al. [32] found that by controlling the degree of cross-linking of EUG, the low-temperature cracking resistance and high-temperature rutting resistance of asphalt could be improved, indicating the feasibility of using SEUG as a modifier in asphalt. Li et al. [33] grafted maleic anhydride onto EUG and then mixed it with asphalt and dry rubber powder. Mixing the EUG-grafted rubber powder with bitumen resulted in a better mix. And the grafting of EUG can improve the compatibility between rubber powder and bitumen. Deng et al. [34] modified rubber asphalt by grafting EUG, and cross-linked chemical bonds were formed between EUG and asphalt. The results showed that grafted EUG could improve the elasticity, viscosity, softening point, rutting resistance, and storage stability of rubber-modified asphalt. Chao et al. [35] showed that the melt method and solvent method of grafting two grafting methods grafting of eucommia ulmoides gum modified rubber powder can be obtained more than ordinary rubber powder. Rubber modified asphalt test performance is better than the solvent method compared to the melt method, and the maleic anhydride grafting rate can be increased by about two times. the rubber asphalt high- and low-temperature performance has been improved. Yu et al. [36] will be prepared to obtain the dulcimer rubber-modified asphalt and the Xinjiang Karamay region asphalt mixture comparison, which found that the dulcimer rubber-modified asphalt has a better resistance to high temperature and a low-temperature cracking performance. And through the two types of asphalt mixture crack extension, the core sample performance and pavement bending and sinking test verified that the dulcimer modified asphalt pavement has better comprehensive performance for the poor quality of asphalt pavement in Xinjiang region and can provide a feasible way for improvement. Li et al. [37] investigated the changes in the properties of the prepared sulfurised dulcimer-modified asphalt through indoor tests. The results showed that with an increase in SEUG doping, the rutting resistance and cracking resistance of the asphalt improved, and the optimum doping of SEUG was proposed to be 10 wt%. Li et al. [38] used a rotational viscosity experiment, a dissociation experiment, a fluorescence microscope, a scanning electron microscope, and other experimental methods to study the performance of dulcimer rubber and rubber-blended and rubber-modified asphalt, and found that dulcimer rubber and rubber powder have good compatibility. The blending process is based on the physical reaction. Dulcimer rubber sulfurization is achieved by the desulfurization of rubber powder. Compared with single rubber-modified asphalt, the blended viscosity and storage stability of rubber-modified asphalt are significantly improved.

At this stage, the gutta-percha-modified asphalt mainly adopts experimental means to study the preparation process, comprehensive performance, and other aspects. Few studies have investigated the compatibility of gutta-percha and asphalt from the molecular scale and the interaction between gutta-percha molecules and asphalt molecules’ microscopic modification mechanism. Based on this, this study intends to use the combination of molecular dynamic simulation and experimental technology at the molecular level This is an in-depth study of EUG- and SEUG-modified asphalt, examing the best preparation process and conducting comparative analysis of EUG, SEUG, and asphalt compatibility. We also study the macroscopic performance of gutta-percha-modified asphalt and profoundly reveal the modification mechanism of gutta-percha-modified asphalt.

To this end, this study first constructed and validated the molecular model of matrix asphalt and then constructed the model of EUG, the molecular model of SEUG with 60% cross-linking degrees, and the model of eucommia gum-modified asphalt with EUG or an SEUG blend of 5 wt%, 10 wt%, and 15 wt%, respectively. Molecular dynamic calculations were performed at 105 °C, 120 °C, 135 °C, 150 °C, 165 °C, 180 °C, and 195 °C to obtain the solubility parameters, intermolecular potential energy, diffusion coefficients, and mechanical property parameters of the stabilized gutta-percha-modified asphalt models, respectively. The compatibility of EUG or SEUG with asphalt and the mechanism of gutta-percha-modified asphalt were discussed in detail. Finally, the reliability of the molecular dynamic simulation was verified by performing conventional performance tests, storage stability tests, and SEM tests on gutta-percha-modified asphalt at different shear temperatures.

This study combines microscopic simulations and macroscopic tests to propose the best preparation methods for EUG-modified asphalt and SEUG-modified asphalt and explores the compatibility between gutta-percha and asphalt, as well as the mechanism of gutta-percha-modified asphalt, providing a certain academic research reference for the development of gutta-percha as a new natural polymer modifier.

## 2. Materials and Methods

### 2.1. Materials and Preparation Process

#### 2.1.1. Original Materials

The EUG used in this study was produced by Xiangxi Laodai Biological Company Limited at Yongshun in China, and its main technical performance parameters are shown in Table 1.

According to the previous research results of our group [42], the promoters used in this study were sulfur supplied by Sinopharm Chemical Reagent Company Limited at Shanghai in China; zinc oxide supplied by Shanghai Jiuqing Chemical Company Limited at Shang-hai in China; stearic acid supplied by Guangdong Xinrunhao Chemical Company Limited at Guangzhou in China; accelerator CZ supplied by Henan Longji Chemical Company Limited at Puyang in China; nano-silica supplied by Hubei Huifu Nanomaterials Company Limited at Yichang in China; naphthenic oil supplied by Jinan Guoxuan Trading Company Limited at Jinan in China; and epoxy resin supplied by Guangzhou Ou Di Chemical Company Limited at Guangzhou in China.

The base asphalt (BA) used in this study was Liaohe A−90 road petroleum asphalt produced by Liaoning Panjin Or Petrochemical Co. Ltd. at Panjin in China, and the technical specifications are given in Table 2.

#### 2.1.2. Preparation Process

(1) The preparation process of vulcanized EUG

According to the previous research results of our group [20], the SEUG preparation process is as follows. First, the double roller opener is preheated to 70–80 °C and held for 5 min, and then a certain calculated amount of EUG is added in batches. After the EUG is heated, softened, and mixed evenly, the distance between the double rollers is increased, and zinc oxide, ZA, nano-silica, and naphthenic are added, and then a certain amount of accelerator CZ and sulfur are added and mixed at about 70 °C for 3–5 min. The samples were placed on a plate vulcanizer for vulcanization, with the temperature, pressure, and time set to 150 °C, 120 MPa, and 35 min, respectively. And it is processed into powder form, ready for use.

(2) The preparation process for the preparation of EUG-modified asphalt

Similarly, the base asphalt is heated and softened to a flowing state and then placed under a high-speed shear emulsifier using an electric heater to heat the base asphalt. The rotor speed was set at 3000 r/min, and the temperature was 150 °C. After 10 min of shearing and mixing, a certain calculated amount of pre-formed powdered EUG was added in batches at an interval of 2 min. The rotor speed was slowly increased to 5000 r/min, and the mixing temperature was set at 165 °C. At this temperature, shearing and mixing were carried out for 1 h until the EUG was uniformly distributed in the bitumen and was kept in an oven. The samples were prepared in a 163 °C oven for 2 h to obtain the EUG-modified bitumen.

(3) The preparation process for the preparation of SEUG-modified asphalt

**Figure 1:**
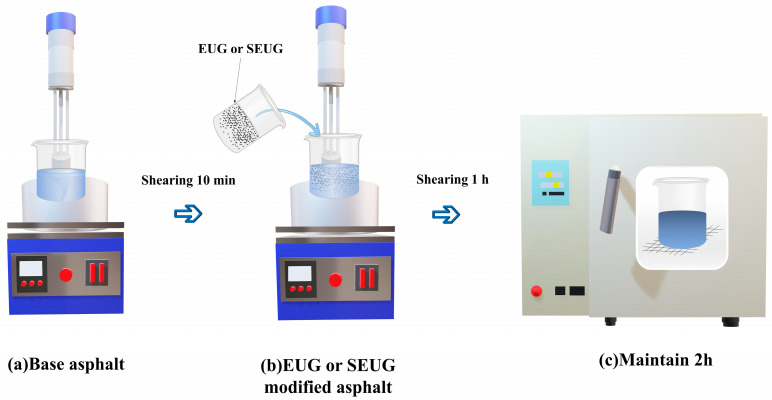
Gutta-percha-modified asphalt process.

The base asphalt is heated and softened to a flowing state and then placed under a BME 100 L laboratory shear while the matrix asphalt is heated at the bottom by an electric heater. The rotor speed is set at 3000 rpm and the temperature is 150 °C. After 10 min of shearing and stirring, a calculated amount of powdered sulfated dulcimer is added in batches at 2 min intervals. Slowly increase the rotor speed to 5000 rpm and set the mixing temperature to 180 °C. Shearing and stirring are carried out at this temperature for 1 h until the SEUG is uniformly distributed in the bitumen. Samples were prepared with 5, 10, and 15 wt% EUG- and SEUG-modified asphalt, as shown in Figure 1.

### 2.2. Experiment Methods

#### 2.2.1. Physical Properties Test

In accordance with the “Technical Specification for Highway Asphalt Pavement Construction (JTG E20–2011)”, the gutta-percha-modified asphalt was tested for its conventional properties to study the effects of mixing temperature and modifier content on the conventional physical properties of asphalt and improve the storage stability of asphalt.

#### 2.2.2. Segregation Experiment

**Figure 2:**
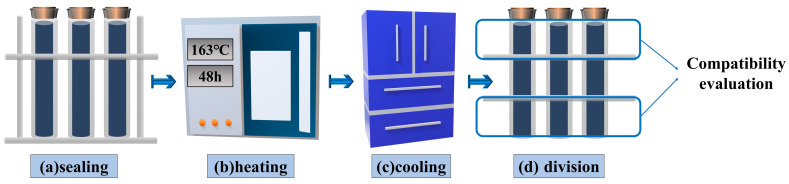
Storage stability tests on gutta-percha-modified bitumen.

In accordance with JTG E20–2011, the storage stability test was carried out on the prepared gutta-percha-modified bitumen, see Figure 2, in a refrigerator for more than 4 h. After freezing, the samples were equally cut into 3 sections. The difference in the softening point between the top and bottom was determined separately to evaluate the storage stability of the gutta-percha-modified asphalt.

#### 2.2.3. Scanning Electron Microscope (SEM)

**Figure 3:**
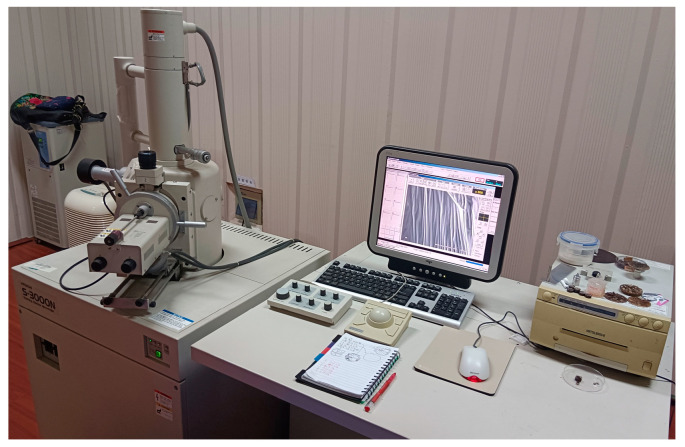
SEM test on gutta-percha-modified bitumen.

In this study, a scanning electron microscope (S−3000N, HITACHI, Hitachi, Japan) was used to observe the effect of different dosages of EUG and SEUG on the micromorphology of asphalt samples. As both gutta-percha and bitumen are non-conductive, all samples were dried in a carbon dioxide critical point dryer (HCP−2, Hitachi, Japan), sprayed with pure gold using a surface treatment machine (SBC−2, China Sciences Group, Beijing, China), and finally observed by SEM to obtain the microstructural characteristics of the samples after magnification 500 times. The test procedure is shown in Figure 3.

### 2.3. Construction of the Molecular Models

#### 2.3.1. Molecular Model of BA

Bitumen is a compound made up of thousands of complex components, consisting mainly of hydrocarbons and functional groups, such as sulfur, nitrogen, and oxygen, making it difficult to accurately describe its chemical structure and predict its properties.

**Figure 4:**
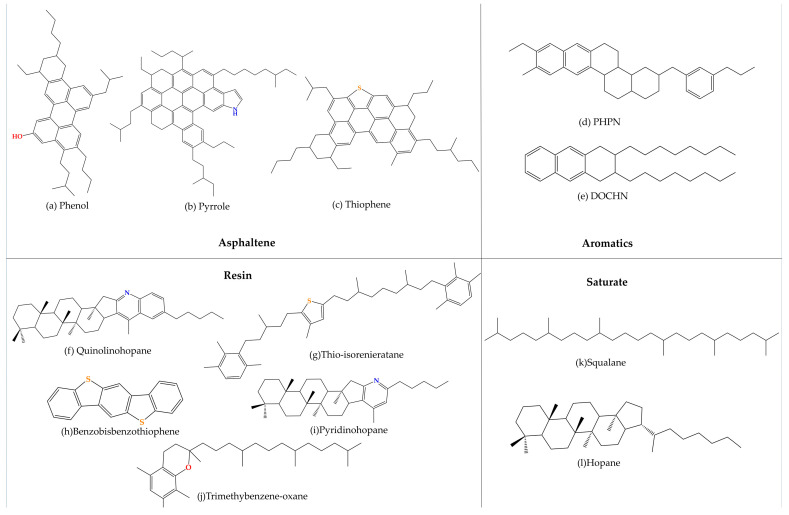
Representative molecules of asphalt molecular models: (**a**–**c**) asphaltene molecules; (**d**,**e**) aromatic molecules; (**f**–**j**) resin molecules; and (**k**,**l**) saturate molecules. Molecules (**a**–**l**) are all from the AAA−1 model [46].

To facilitate the analysis of asphalt composition, Corbett [44] proposed a four-component analysis method (SARA), which classifies asphalt into saturate, aromatic, resin, and asphaltene. According to the AAA−1 model (later abbreviated as AAA−1), a molecular dynamic model for asphalt proposed by Li and Greenfield [45], asphalt can be further subdivided into 12 components, and the specific molecular composition information in the AAA−1 model is shown in Figure 4.

**Figure 5:**
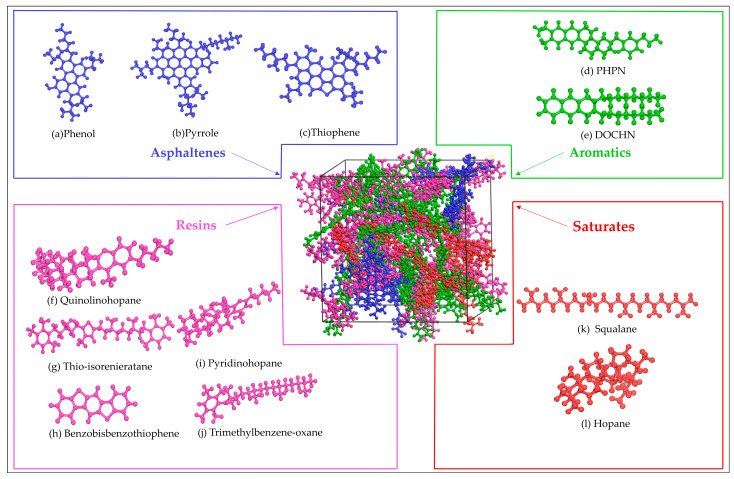
The 12-component MD model of asphalt: the blue color refers to asphaltene, the green color refers to aromatics, the pink color refers to resin, and the red color refers to saturate.

In order to reasonably reduce the computational workload, the number of atoms in the matrix asphalt model in this study is proposed to be controlled between 5000 and 10,000. The ratio of the four components of the AAA−1 model was found to be similar to Liaohe A−90 road bitumen, indicating that the AAA−1 model can be used to simulate Liaohe A−90 road bitumen. In the Materials Studio software (Materials Studio version 2005), the matrix asphalt model was built, as shown in Figure 5, according to Table 3.

#### 2.3.2. Molecular Model of EUG

**Figure 6:**
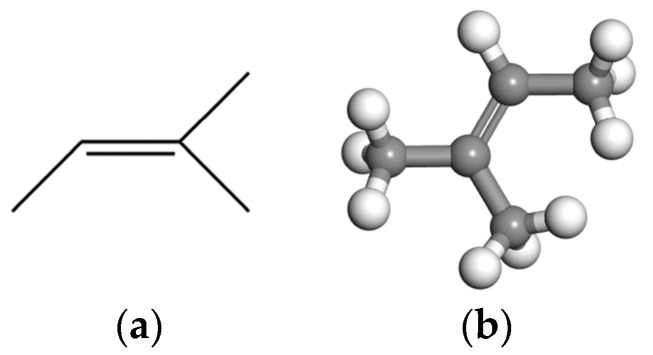
Molecular structure fluxes of EUG: (**a**) molecular structure fluxes of EUG; (**b**) EUG model.

**Figure 7:**
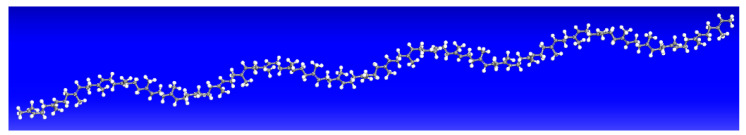
Single-chain molecular model of EUG with *N* = 30.

EUG is an off-white solid particulate material and a very important periodic chain hydrocarbon polymer, whose main chemical component is trans−1,4-polyisoprene. The molecular structure formula and monomer model of EUG are shown in Figure 6a,b. For the minimum degree of polymerization of EUG, the study by our group showed that *N* = 30 is the minimum degree of polymerization of EUG [42], and this study will directly apply this conclusion for the construction of the single-chain model of EUG. The established molecular model of EUG is shown in Figure 7.

#### 2.3.3. Molecular Model of SEUG

**Figure 8:**
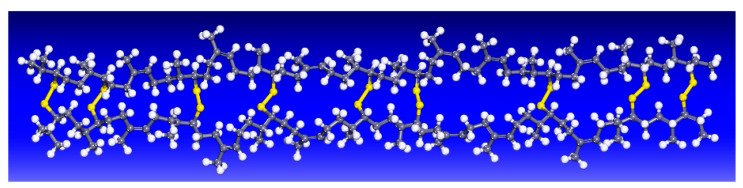
Molecular model of SEUG consisting of 100 SEUG monomers cross-linked with C-S-S-C bonds: yellow color represents a double sulfur bond.

The molecular model of SEUG was further developed based on the molecular model of EUG. It has been shown that the suitable cross-linking degree of SEUG should be controlled at 40~80% [42]. Therefore, in this study, a molecular model of SEUG with a cross-linking degree of 60% was selected for construction, and the Materials Visualizer module was used to construct a single-chain model of SEUG with a 60% cross-linking degree using a sulfur bridge (C-S-S-C) as the cross-linking bond, as shown in Figure 8, where the cross-linking degree (*DC*) was defined as in Refs. [48,49,50] and calculated by following equation:

(1) $$DC=\frac{2N_{CL}}{N_{mono}}\cdot 100\%$$

where *N_CL_* denotes the total number of cross-linked bonds and *N_mono_* denotes the number of monomers.

#### 2.3.4. Molecular Model of Gutta-Percha-Modified Asphalt

**Figure 9:**
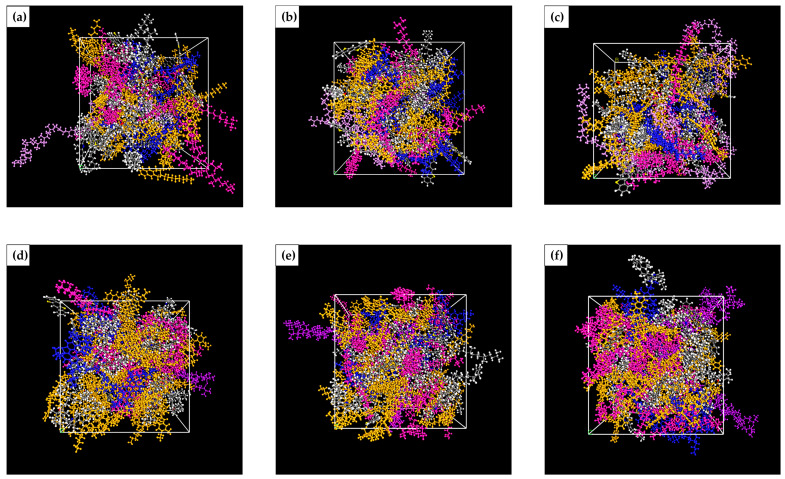
Molecular modeling of modified asphalt with different modifier contents: (**a**) 5 EA; (**b**) 10 EA; (**c**) 15 EA; (**d**) 5 SA; (**e**) 10 SA; (**f**) 15 SA.

**Figure 10:**
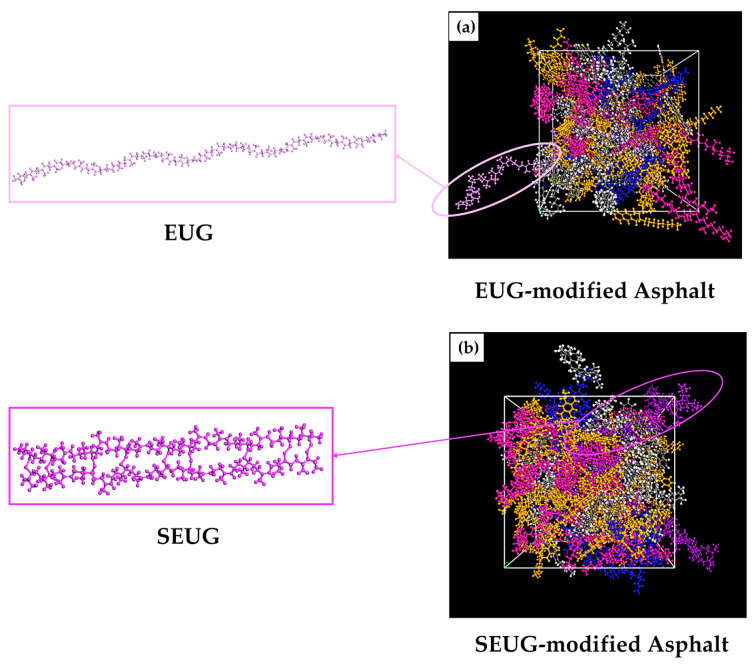
Enlarged partial view of EUG- and SEUG-modified asphalt: (**a**) EUA-modified asphalt; (**b**) SEUG-modified asphalt.

To investigate the effect of the modifier on asphalt performance, different amounts of EUG and SEUG molecules were added to the asphalt using the amorphous cell calculation module to construct different modifier content of EUG-modified asphalt. The modifier mass fractions were taken to be 5%, 10%, and 15%, respectively. Gutta-percha-modified asphalt and the molecular model are shown in Figure 9 and Figure 10. The name of the modified bitumen models, the number of modifier molecules, and the mass fraction of the modifier in the bitumen model are shown in Table 4.

### 2.4. MD Simulation Methods and Task

#### 2.4.1. Theoretical Basis

Molecular dynamic (MD) simulations are based on classical mechanics and mathematical simulation calculations to study the properties of individual molecules or molecular systems or even whole systems and are thus becoming an important visualization tool for the study of the physical properties of complex phenomena and materials. MD simulation methods were first proposed by Wainwright [51] in the 1950s to solve the Newtonian equations of motion for multi-particle systems. After a century of development, they have become the main theoretical methods for studying the properties and principles of the action of microscopic matter.

(1) Equations of Motion

The molecular dynamic approach is based on two assumptions [52,53]: (1) all particle motion processes follow Newton’s classical laws of mechanics; (2) particle motion is governed by the superposition principle and Newton’s equations of motion are used to describe the particle processes, which are calculated as follows [54,55]:

(2) $$a_{i}=\frac{d^{2}r_{i}}{dt^{2}}=\frac{F_{i}}{m}\;\;\;(i=1,2,\ldots n)$$

(3) $$F_{i}=-\frac{\partial U}{\partial r_{i}}$$

where $F_{i}$ is the combined force on the i−th particle; $r_{i}$ is the coordinate vector of the particle; $m_{i}$ is the mass of the particle; and $a_{i}$ denotes the velocity and acceleration of the particle. U denotes the potential energy of the particle, which is calculated by the potential function.

Approximating the forces and potentials between pairs of atoms using potential functions greatly simplifies computer calculations and facilitates the calculation of condensed systems, such as asphalt, on larger scales.

(2) Boundary Conditions

Since it is impossible to precisely determine each molecule’s position in a complex system of condensed nature, like dulcimer and asphalt, which contains a variety of molecules, there is uncertainty in the traversal and boundary effects [56]. Therefore, periodic boundary conditions are frequently used to remove boundary effects. One particle in the model box represents an infinite number of particles at a specific location, effectively eliminating the boundary effect [57]. When periodic boundary conditions are introduced, the simulated system transforms into a central cell, and through the periodic boundary conditions, the image of the central cell repeats periodically in the three-dimensional molecular system.

(3) Force Field

The COMPASS force field in Materials Studio [58] is an ab initio force field consisting of condensed matter properties, various isolated molecules, and empirical data that can accurately predict the structure, vibrational, and thermal properties of isolated or condensed matter systems over a wide range of temperatures and pressures. The applicability, therefore, extends to almost all covalent molecular systems. The COMPASS II force field, developed from the COMPASS force field, has been further improved in terms of generalizability and accuracy, and this study will be followed by molecular simulations and calculations of asphalt models based on the COMPASS II force field [59].

(4) Ensemble

Ensemble is a collection of systems with constant macroscopic conditions and identical properties, each in a different microscopic state and independent of the other, which is also known as a statistical hedge. Of these, the regular ensemble (NVT) and the constant temperature and pressure ensemble (NPT) are the most commonly used hedges for polymer molecular dynamic simulations. The regular ensemble (NVT) is a system in which the total number of particles (N), the total volume (V), and the temperature (T) are kept constant, and fluctuations in the pressure of the system are allowed. In contrast, the isothermal isobaric ensemble (NPT) is a system in which the total number of particles (N), the pressure (P), and the temperature (T) remain constant, and fluctuations in the system energy E and the system volume V are possible. This study will be followed by molecular dynamic calculations of the bitumen model for the NVT and NPT systems [59].

#### 2.4.2. Simulation Process

**Figure 11:**
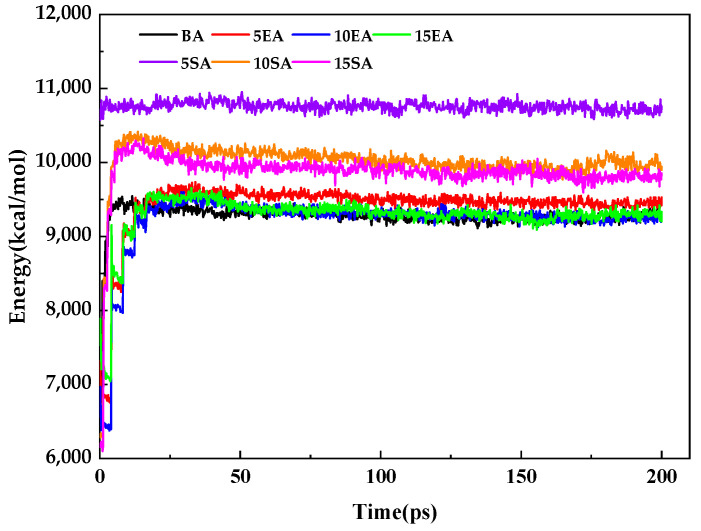
Energy variation of different asphalt models with results coming from NPT after 200 ps volume shrinking by NVT.

Molecular dynamic simulations of the constructed molecular model of different asphalt were performed as follows:

(1) Due to the very high initial energy of the bitumen system, a structural optimization of the bitumen model is required to find the best local energy point before molecular dynamic simulations can be performed. This is performed by using the geometry optimization module of the forcite module for geometric optimization, selecting the smart algorithm, setting the maximum number of iterations to 50,000, selecting an accuracy of medium, a truncation radius of 12.5 Å, a force field from COMPASS II, and using the atom-based and ewald methods, respectively. The van der Waals non-bonded and electrostatic non-bonded interactions were solved with the charge set to forcefield.
(2) Annealing allows the bitumen molecular chains to relax, the cell volume to decrease, and the model densities to increase, thus eliminating the local energy minimum of the system. This brings the bitumen model closer to the natural molecular state of the bitumen. Annealing of the bitumen model was carried out using the anneal task in the forcite module with a constant temperature and pressure system (NPT) set at 27–1527 °C, 5 cycles of temperature rise and fall, and a total simulation time of 200 ps.
(3) The premise of the system calculation is that the system is in thermodynamic equilibrium; so, the asphalt model in this study was subjected to a time step of 1 fs and had a total simulation time of 200 ps for the constant volume system (NVT) calculation and 200 ps for the constant pressure system (NPT) kinetic calculation. According to existing studies [60], thermodynamic parameters, such as energy, change by 5~10% as the simulation time increases, and the system is assumed to reach a steady state. The simulation results are shown in Figure 11.

In Figure 11, it can be seen that the system is in a state of dynamic change until the simulation duration is 40 ps, resulting in the violent thermal motion of the molecules, which makes the total energy of the system also in a state of constant change. However, when the simulation duration exceeded 40 ps, the thermal motion of molecules began to stabilize and the total energy of the system began to converge, with the variation of the total energy in the range of 1.52 to 2.31%, indicating that the asphalt model reached a steady state at this time.

#### 2.4.3. Simulation Task

The forcite module meter was used to calculate and analyze the solubility parameters, intermolecular interaction energy, diffusion coefficient, and mechanical properties of each asphalt system in order to more clearly describe the interaction between asphalt and gutta-percha.

(a) Solubility parameters

The cohesive energy density (*CED*), which is a physical quantity that characterizes the strength of intermolecular interactions in a substance, is defined by the theory of heat of mixing of polymer blends as the energy required to dissipate all intermolecular forces in 1 mol of a substance. The solubility parameter (*δ*), which can be used as a physicochemical indicator to assess the compatibility of substances, is obtained by squaring the cohesive energy density and is calculated as follows [61]:

(4) $$\Delta H_{n}=\left[\frac{N_{i}V_{i}\cdot N_{j}V_{j}}{N_{i}V_{i}+N_{j}V_{j}}\right]\left[\sigma_{i}-\sigma_{j}\right]$$

(5) $$\delta_{i}=\sqrt{\frac{\Delta E_{i}}{V_{i}}}$$

(6) $$\delta_{j}=\sqrt{\frac{\Delta E_{j}}{V_{j}}}$$

(7) $$\Delta \delta =\left|\delta_{i}-\delta_{j}\right|$$

where *N_i_* and *N_j_* are the relative molecular masses of polymers *i* and *j*, respectively; *V_i_* and *V_j_* are the molar volumes of polymers *i* and *j*, respectively; $\frac{\Delta E_{i}}{V_{i}}$,$\frac{\Delta E_{j}}{V_{j}}$ are the cohesive energy densities of polymers *i* and *j*, respectively; $\delta_{i}$, $\delta_{j}$ are the solubility parameters of polymers *i* and *j*, respectively; and Δδ is the absolute value of the difference between the solubility parameters of polymers *i* and *j*.

(b) Intermolecular potential energy

The molecular bond length and bond angle of each system are constantly changing during the molecular dynamic simulation calculation, and the system’s deformation and distortion are also very complex. The formula for the intermolecular interaction energy, which can be used to thoroughly assess both the system’s stability and the intermolecular interaction, is as follows [62]:

(8) $$E_{p}=E_{jkp}-E_{jp}-E_{kp}$$

(9) $$E_{V}=E_{jkV}-E_{jV}-E_{kV}$$

(10) $$E_{\varepsilon }=E_{jk\varepsilon }-E_{j\varepsilon }-E_{k\varepsilon }$$

where $E_{p}$ is the molecular potential energy; $E_{jkp}$, $E_{jp}$, and $E_{kp}$ are the molecular potential energies of polymers *jk*, *j*, and *k*, respectively; $E_{V}$ is the van der Waals potential energy; $E_{jkV}$, $E_{jV}$, and $E_{kV}$ are the van der Waals potential energies of polymers *jk*, *j*, and *k*, respectively; $E_{\varepsilon }$ is the electrostatic potential energy; and $E_{j\varepsilon }$ and $E_{k\varepsilon }$ are the electrostatic potential energies of polymers *jk*, *j*, and *k*, respectively.

(c) Diffusion coefficient

The phenomenon of diffusion is derived from the movement of particles in space. By studying the inter-diffusion of gutta-percha particles in bitumen, the active degree of mutual movement of gutta-percha particle molecules and bitumen molecules at a given temperature can be analyzed, thus providing good conditions for the mixing of gutta-percha and bitumen. Therefore, the aim of this study is to investigate the dispersion and migration ability of the modifier in bitumen using the mean square displacement (*MSD*) as well as the diffusion coefficient (*D*), where the *MSD* is calculated as:

(11) $$MSD(t)=\langle {\left|r(t)-r(0)\right|}^{2}\rangle$$

where 〈 〉 denotes the average value for all particles in the group and *r*(*t*) denotes the displacement vector of the particles in the group at time *t*.

According to the rise and fall dissipation theory in non-equilibrium statistical thermodynamics, 1/6 of the slope of the applied *MSD* diffusion phase curve is the diffusion coefficient *D* [63].

(12) $$D=\underset{t\to \infty }{\lim}\frac{{\left|r(t)-r(0)\right|}^{2}}{6t}=\underset{t\to \infty }{\lim}\frac{MSD(t)}{6t}=\frac{m}{6}$$

where *D* is diffusion coefficient; *t* is time (ps); *r*(*t*) is the coordinate of the molecular (Å^2^); and *m* is the slope of the *MSD* curve.

(d) Mechanical performance calculations

Mechanical properties are the ability of a polymer to resist deformation when subjected to external forces and have a very important influence on the preparation, processing, and application of the polymer. The mechanical properties module of forcite can be used to calculate the Larmé constants *λ* and *μ* for the base asphalt and the gutta-percha-modified asphalt when the structure is stabilized. We use the Larmé constants, and the parameters for the mechanical properties of the gutta-percha-modified asphalt can be calculated as [64,65]:

(13) $$K=\lambda +\frac{2}{3\mu }$$

(14) G=μ

(15) $$E=\frac{\mu \left(3\lambda +2\mu \right)}{\lambda +\mu }$$

(16) $$\nu =\frac{\lambda }{2\left(\lambda +\mu \right)}$$

where *K* is the bulk modulus; *G* is the shear modulus; *E* is Young’s modulus; and *ν* is the Poisson’s ratio.

*E* is an important indicator of the stiffness of a material; the higher the value, the greater the stiffness and resistance to deformation of the material. *G* is used to assess the material’s resistance to shear deformation. *K* is used to describe the incompressibility and elasticity of a material. *ν* is used to measure the resistance of a material to transverse deformation.

## 3. Results and Discussion

### 3.1. Molecular Model Validation

**Figure 12:**
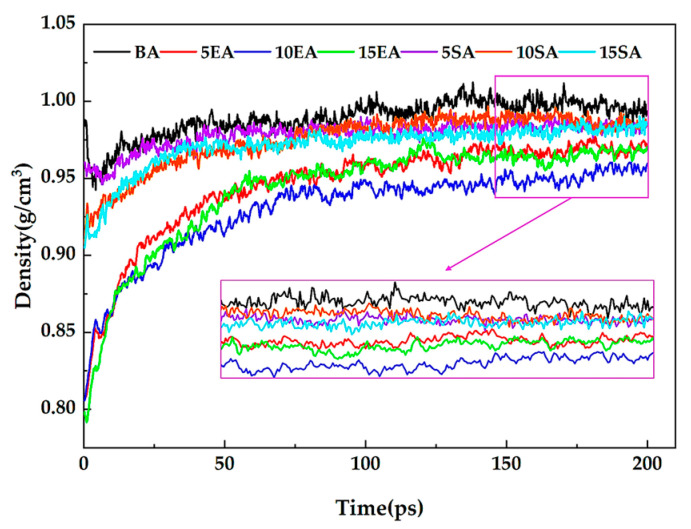
Simulation values of density after NPT for different asphalt models.

**Figure 13:**
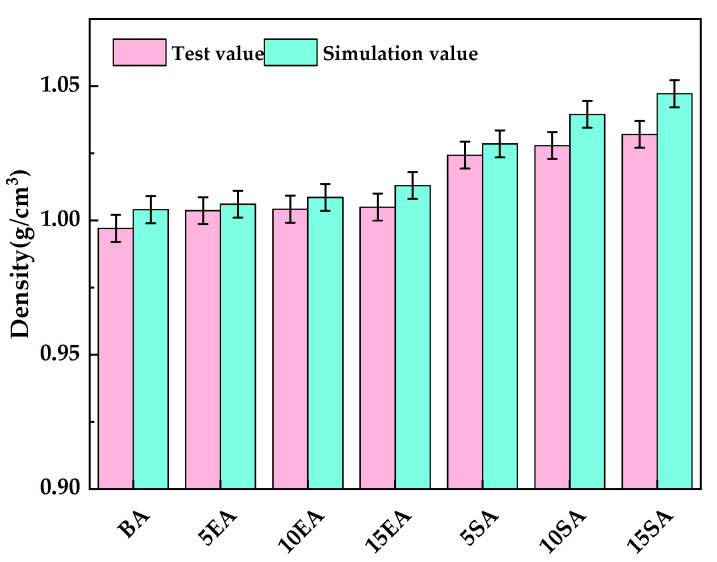
Comparison of the simulation and test values of different modified asphalt models.

The modified asphalt model can be used to verify the validity of the molecular model through density, which is one of the key thermodynamic parameters of asphalt [66]. Figure 12 displays the densities of the various asphalt models that were obtained after the MD simulation. As the simulation time length increases, the densities of the various asphalt models converge and eventually reach stability, indicating that the model is continuously converging toward thermodynamic equilibrium. This study chose a simulation time of 150~200 ps, reached the dynamic equilibrium of various asphalt systems using molecular models, and then took the average of the simulated values for density to ensure the accuracy of the results. Figure 13 displays the comparison between the measured values and the simulated values. The simulation values of various asphalt models are very similar to the test values, as shown in Figure 13, and the difference between the simulated and measured values of the density for all asphalt models is calculated to be less than 2%. This finding demonstrates the validity of the molecular model.

### 3.2. Simulation Results and Discussion

#### 3.2.1. Solubility Parameters

**Figure 14:**
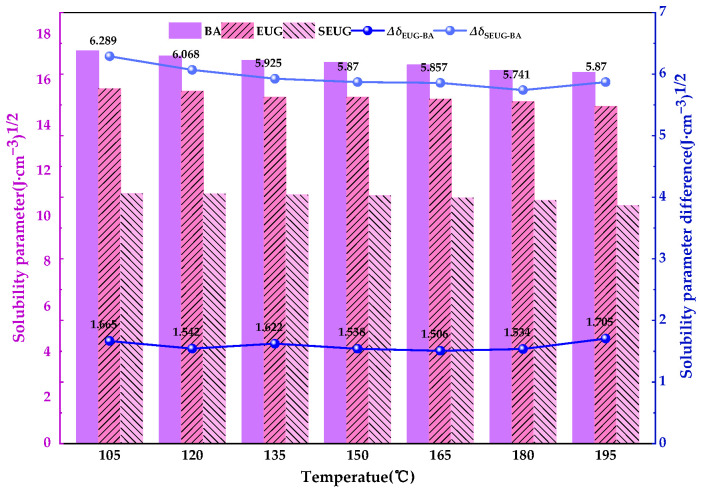
Solubility parameters and differences in solubility parameters between BA and the modifier.

We use the cohesive energy density task. Using forcite, the solubility parameters of each molecular model can be calculated. The smaller the difference between the solubility parameters of the different molecular models and their solubility parameters, the better the compatibility of the two substances. The solubility parameters and solubility parameter differences between the matrix bitumen and the two types of EUG are shown in Figure 14.

**Figure 15:**
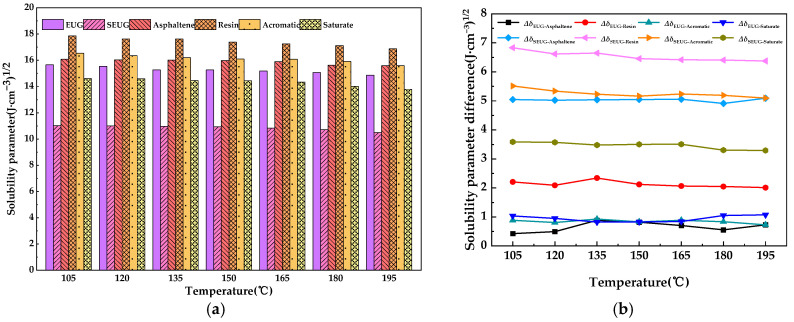
Solubility parameters and solubility parameter differences between the four BA components and modifiers: (**a**) solubility parameter; (**b**) solubility parameter difference.

As can be seen in Figure 14, the solubility parameters of both the bitumen model and the two types of EUG models show a decreasing trend with increasing temperature. This is due to the fact that as the temperature increases, the absorption of thermal energy by the molecules also increases, resulting in more violent irregular molecular motion, higher kinetic energy, larger macroscopic volume, and lower density of molecular cohesion energy, leading to a decrease in solubility parameters. Furthermore, as can be seen in Figure 14, the overall difference in solubility parameters between bitumen and EUG appears to be much smaller than the difference in solubility parameters between bitumen and SEUG, indicating that EUG is more compatible with bitumen than SEUG is with bitumen, and the difference in solubility parameters between bitumen and EUG reaches its minimum at 165 °C and 1.506 (J·cm^−3^)^1/2^, indicating that bitumen and EUG were most compatible at 165 °C. To further analyze the compatibility of the four components of EUG and bitumen, the dissolution parameters of the two EUG models with the four components of bitumen and the difference in dissolution parameters were studied and analyzed separately, as shown in Figure 15a,b.

As can be seen in Figure 13a, among the constituents of bitumen, the solubility parameters of asphaltene and resin are high, while the solubility parameters of aromatic and saturate are relatively low. Most of the asphaltene and resin are composed of polycyclic aromatic structures based on benzene rings, while most of the saturate and aromatic are composed of carbon-based alkanes. The vulcanization process causes a significant change in the molecular structure of gutta-percha from linear to reticulated. Thus, based on the reactive properties of structural homology, EUG is naturally close to asphaltene and resin, while SEUG is correspondingly close to saturate and aromatic. It can also be seen in Figure 13b that the difference in solubility parameters between EUG and asphaltene and resin in bitumen is significantly smaller than the difference in solubility parameters between EUG and saturated and aromatic fractions; the difference in solubility parameters between SEUG and saturate and aromatic in bitumen is also significantly smaller than the difference in solubility parameters between SEUG and asphaltene and resin. This indicates that the compatibility of EUG with asphaltene and resin is better than EUG with the other two components, while the opposite is true for SEUG, which is more compatible with saturate and aromatic than SEUG with the other two components.

#### 3.2.2. Intermolecular Potential Energy

The energy task in the forcite module was used to calculate the intermolecular potentials of the various asphalt systems. The outcomes are displayed in Table 5 of this publication.

**Figure 16:**
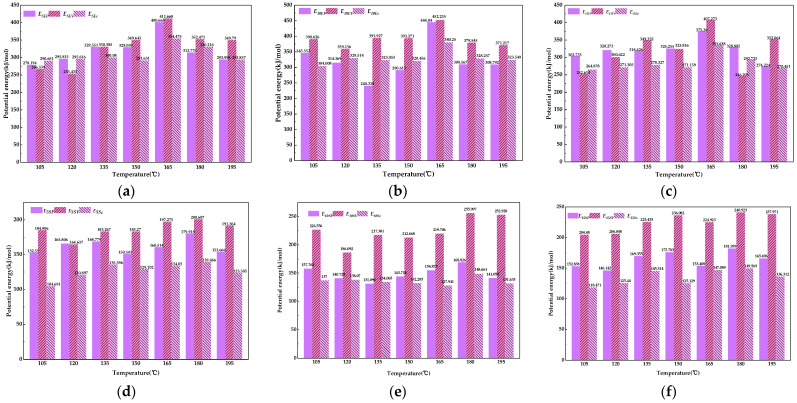
Molecular potential energy between asphalt and different content of the modifier: (**a**) 5 EA, (**b**) 10 EA, (**c**) 15 EA, (**d**) 5 SA, (**e**) 10 SA, (**f**) 15 SA.

According to Table 5, using Equations (6)–(8), the *E_V_*, *E_P_*, and *E_ε_* between the modifier molecules and asphalt molecules in the matrix asphalt and each gutta-percha-modified asphalt can be seen in Table 4. When the distance between molecules exceeds the equilibrium position, the molecular potential energy is negative, and after taking the absolute value for it, the relationship between molecular potential energy and temperature can be obtained, as shown in Figure 16a–f.

As can be seen in Figure 16, the temperature has a significant effect on the molecular, van der Waals, and electrostatic potential energies between EUG and bitumen and SEUG and bitumen. In the temperature range of 105 to 195 °C, each interaction energy shows an increasing and then decreasing trend. The increase in the intermolecular potential energy means that the hybrid system of EUG and bitumen becomes more stable, and there is a difference in the fluctuation of the molecular potential energy between the two hybrid systems of EUG, SEUG, and bitumen.

The intermolecular potential energy between bitumen and 10 EA peaks around 165 °C, indicating that at 165 °C, the 10 wt% content of EUG is more likely to depolymerize with bitumen, resulting in the best compatibility between EUG and bitumen and the most stable structure of EUG-modified bitumen. This conclusion is consistent with that obtained from the solubility parameter analysis.

#### 3.2.3. Diffusion Coefficient

**Figure 17:**
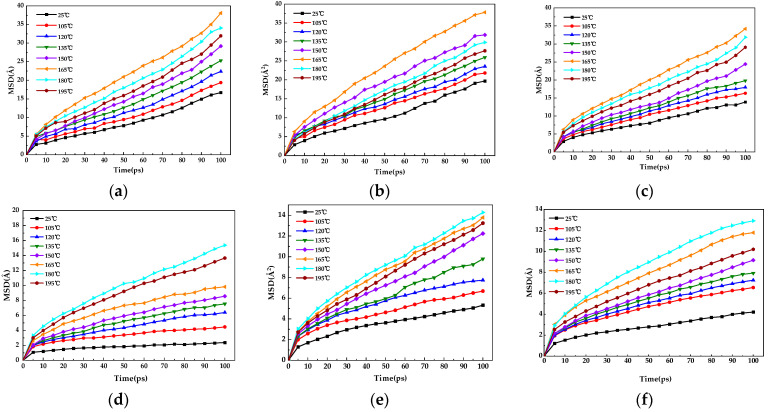
MSD of modified asphalt with different content of the modifier as a function of temperature: (**a**) 5EA; (**b**) 10EA; (**c**) 15EA; (**d**) 5SA; (**e**) 10SA; (**f**) 15SA.

The mean square displacement task was performed using the analysis module under forcite, and the xtd file of the modified bitumen with gutta-percha generated by the dynamic command was analyzed with EUG and SEUG as the set object to obtain the curve of *MSD* of gutta-percha in bitumen versus time, and the results are shown in Figure 17a–f.

**Figure 18:**
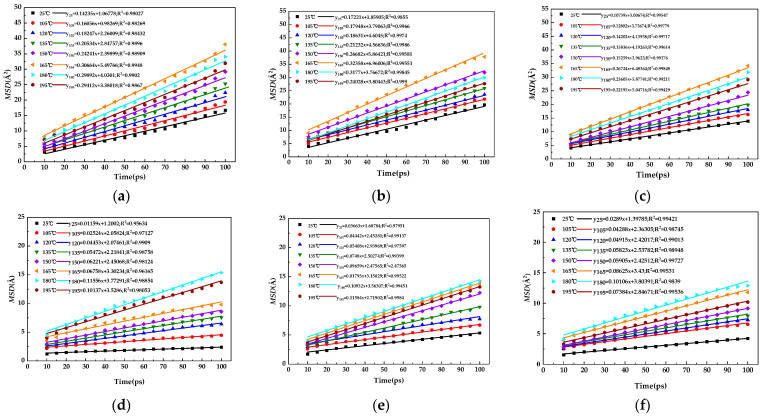
Results of the point-line fit of the *MSD* data for modified bitumen with different content of the modifier: (**a**) 5 EA; (**b**) 10 EA; (**c**)15 EA; (**d**) 5 SA; (**e**)10 SA; (**f**) 15 SA.

As shown in Figure 17a–f, the curve rises rapidly within the first 10 ps, indicating that the gutta-percha and asphalt molecules are rapidly approaching each other with distance at this time; after 10 ps, the diffusion rate tends to stabilize, indicating that the co-mingling of gutta-percha and asphalt is gradually stabilizing. At this point, the gutta-percha molecules and the asphalt molecules would be diffusing at the interface, which is the diffusion stage required for the study. Therefore, the results of the *MSD* data at different simulated temperatures after 10 ps were linearly fitted, and the fitted results are shown in Figure 18a–f.

**Figure 19:**
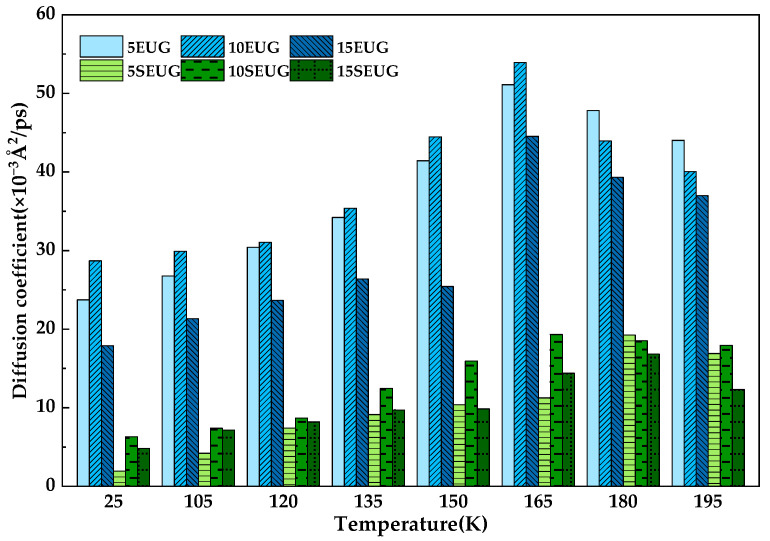
Comparison of the diffusion coefficient modifiers in asphalt.

The slope of the linear fit in Figure 18 was calculated using Equation (10) to obtain the *D*. The diffusion coefficient of the SEUG molecules in bitumen was plotted, as shown in Figure 19. It can be seen in Figure 19 that as the temperature increases, the *D* of both EUG and SEUG in bitumen show a tendency to increase and then decrease. The slope of the linear fit for the EUG-modified asphalt is significantly higher than the SEUG-modified bitumen, indicating that the diffusion of EUG is more intense than SEUG in asphalt due to the fact that cross-linked SEUG is more likely to absorb the light components of the bitumen for the swelling reaction compared to EUG. The diffusion causes the SEUG molecules to become larger and slows down the diffusion of SEUG molecules in the bitumen. Among the three groups of EUG-modified asphalt, the diffusion coefficient of 10 wt% EUG-modified asphalt was the largest and reached a maximum of 16.91 × 10^−10^m^2^·s^−1^ at 165 °C. This indicates that the 10 wt% EUG was the most active at 165 °C, which is also consistent with the findings of the previous study.

#### 3.2.4. Mechanical Performance Calculations

The results of the calculation of the mechanical parameters of different asphalt molecular modules are shown in Table 6. It can be seen that the *E*, *K*, *G,* and *ν* of both EUG-modified bitumen and SEUG-modified bitumen are greater than the matrix bitumen, indicating that both EUG and SEUG are beneficial in improving the mechanical properties of the bitumen. The *E*, *K*, *G,* and *ν* of both EUG- and SEUG-modified asphalt showed a tendency to increase and then decrease with the increase in the modified admixture, with the best mechanical property parameters occurring at 10% of the modified admixture. This is due to the fact that the gutta-percha gum absorbs the light components of the bitumen and achieves a uniform dispersion, which enhances the interaction between the gutta-percha system and the bitumen system and promotes the improvement of the mechanical properties of the gutta-percha-modified asphalt system. However, if the amount of gutta-percha exceeds the optimum amount, the homogeneity of the system will decrease, the mechanical properties will also decrease, the interaction between the two will weaken, and the equilibrium of the system will be disturbed, causing the juniper berries to separate from the asphalt. As the amount of gutta-percha added is further increased, the effect of the particles on the modulus of the modified asphalt system will exceed the intermolecular interaction, resulting in a similar aggregate. The “filling” phenomenon will further improve the mechanical properties of the system, but at this point, the system is still in a state of instability. Therefore, when selecting gutta-percha, its stability and mechanical properties in asphalt must be fully considered. Under the premise of meeting the stability of the system, the mechanical properties of a good SEUG admixture of 10 wt%, when the SEUG-modified asphalt was higher than the *E*, *K*, *G*, and *ν* of BA, were 28.6, 22.51, 28.13, and 15.62%.

### 3.3. Experimental Results and Discussion

#### 3.3.1. Physical Properties Test

**Figure 20:**
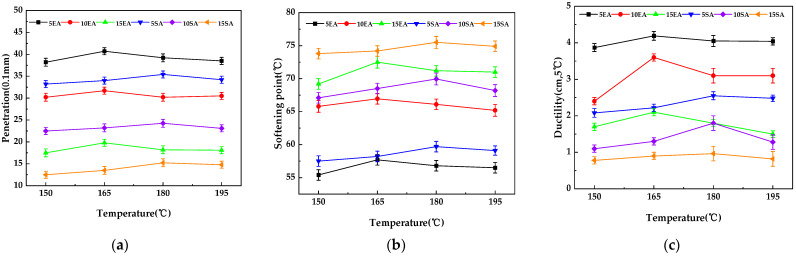
Physical properties of the EUG/SEUG-modified asphalt at different production temperatures: (**a**) penetration; (**b**) softening point; (**c**) ductility.

The physical properties of the gutta-percha-modified asphalt at different processing temperatures are shown in Figure 20a–c. It can be seen in the figures that the three main indicators (penetration, softening point, and ductility) of the gutta-percha-modified asphalt show an increasing and then decreasing trend, and the peak of the curve of the three indicators of the EUG-modified asphalt corresponds to a temperature of 165 °C. The peak of the curve of the three indicators of the SEUG-modified asphalt corresponds to a temperature of 180 °C, indicating that the best compatibility temperature of the EUG-modified asphalt is 165 °C compared to other preparation temperatures.

**Figure 21:**
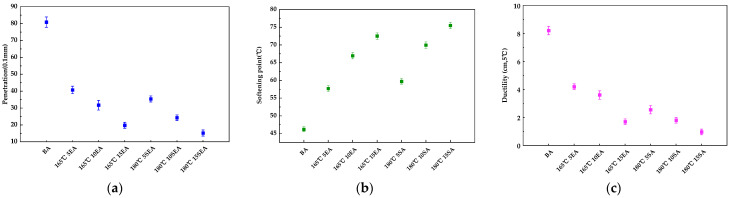
Experimental results on the three main indicators of gutta-percha-modified asphalt with different modifier content: (**a**) penetration; (**b**) softening point; (**c**) ductility.

The physical properties of the gutta-percha-modified asphalt with different modifier blends at the optimum formulation temperature are shown in Figure 21a–c. As can be seen in Figure 21, the penetration and ductility of the EUG- or SEUG-modified asphalt are significantly lower than the base asphalt, and the softening point is significantly higher than the base asphalt. This indicates that the addition of both EUG and SEUG improves the high-temperature performance of the bitumen, but also reduces the low-temperature performance of the bitumen. Furthermore, the penetration and ductility of the EUG-modified asphalt are higher than the SEUG-modified asphalt, but the softening point is lower than the SEUG-modified asphalt, indicating that SEUG is more important than EUG in improving the high-temperature performance of the bitumen. This can be attributed to the fact that SEUG is more prone to swelling in the bitumen than EUG, resulting in a loss of saturate and aromatic, a higher proportion of asphaltene and resin, and a higher stiffness of the modified asphalt, leading to changes in the three main indicators. This conclusion is also consistent with the results of the mechanical property simulations.

#### 3.3.2. Segregation Experiment

**Figure 22:**
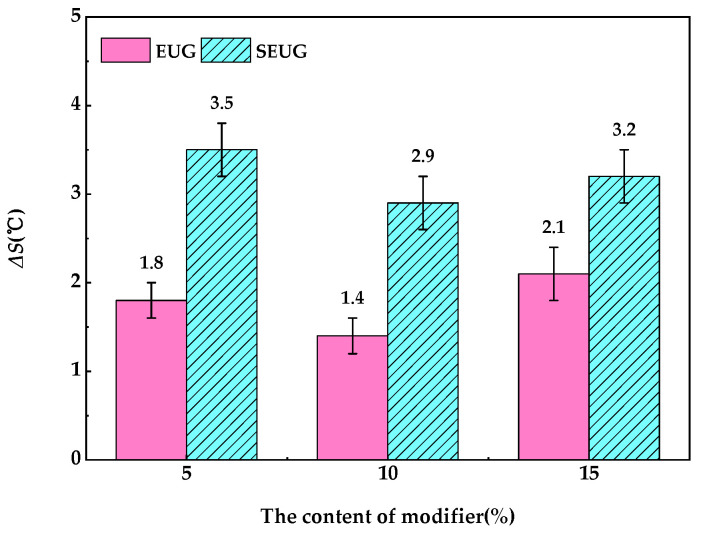
Storage stability test results of gutta-percha-modified asphalt.

Figure 22 shows the results of the segregation test of the EUG/SEU-modified bitumen at the optimum processing temperature. As can be seen in Figure 20, the difference in the softening point (Δ*S*) of the two types of gutta-percha-modified bitumen tended to decrease with increasing EUG or SEUG addition and then increased after 48 h. This is due to the compatibility effect reduced by the polarity difference between EUG and bitumen. In addition, after 48 h at high temperature, the light component in the bitumen was absorbed by the gutta-percha, resulting in a higher density of the gutta-percha. When the density of the EUG was greater than the bitumen, the gutta-percha precipitated and sank in the lower layer of the aluminum tube under the effect of gravity, and the asphalt in the upper layer of the aluminum tube showed an increase in the softening point due to a decrease in the proportion of the light component and an increase in the proportion of recombination. In addition, the ΔS of EUG-modified asphalt was 1.1–1.7 °C higher than SEUG-modified asphalt, and the minimum values of ΔS all occurred at 10 wt%, which is also consistent with the previous simulations. This indicates that EUG has better high-temperature storage stability than SEUG. Combined with the analysis of the simulation results, the hydrogen bonding and van der Waals forces between EUG content and bitumen are strongest at 10 wt%, which makes EUG fully compatible with asphalt, and EUG-modified asphalt has excellent storage stability.

#### 3.3.3. SEM Test

**Figure 23:**
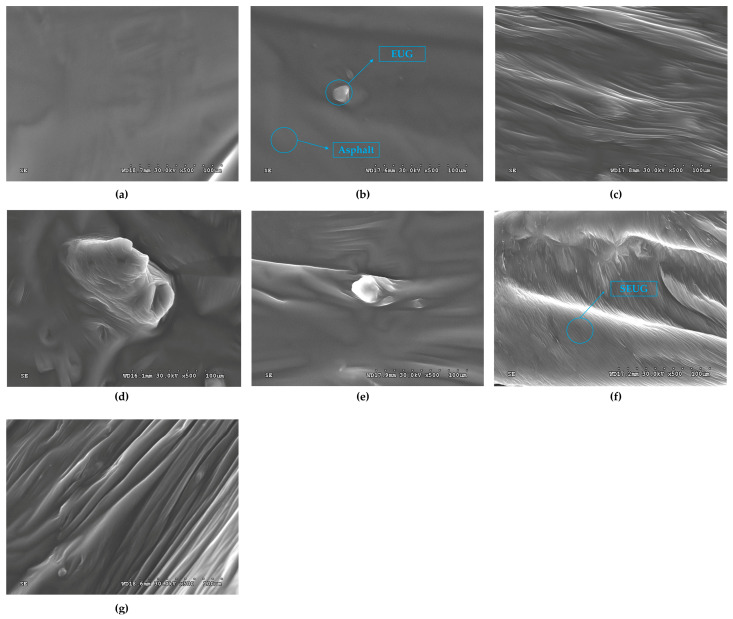
SEM test results of different dosages of gutta-percha-modified asphalt amplified 500 times: (**a**) BA; (**b**) 5 EA; (**c**) 10 EA; (**d**) 15 EA; (**e**) 5 SA; (**f**) 10 SA; (**g**) 15 SA.

SEM was used in this study to look into how the gutta-percha affected the micromorphology of asphalt. Figure 23a–g display the results of the SEM test on base asphalt, modified asphalt with SEUG, and modified asphalt with various dosages of the gutta-percha.

The BA sample has a smooth, flat surface that is homogeneous in structure, as shown in Figure 23a.

Figure 23b–d show that, overall, it looks like EUG-modified asphalt has a non-homogeneous structure and that EUG is mostly present as granular deposits that are encased in asphalt. In Figure 23b, the surface of the 5 wt% EUG-modified asphalt sample is relatively flat; there are only a few EUG particles present, and asphalt dominates the sample as a whole, indicating that there are not enough EUG drugs. In Figure 23c, EUG particles are uniformly coated by asphalt, and uniform wrinkles appear on the surface of modified asphalt, suggesting that high-temperature and high-speed shear will promote a small portion of EUG from the plastic state to the rubber state of the transition. But because there is no sulfurizing agent present, EUG is still primarily in the plastic state in asphalt, and the uniform distribution of EUG suggests that at this time, the EUG is in mixing mode. The fact that the EUG particles in Figure 23d were clearly agglomerated and formed an unevenly sized raised and concave structure suggests that the modifier dosage at this time was too high, making it impossible for the EUG to be evenly dispersed in the asphalt.

As seen in Figure 23e–g, SEUG seems to be less compatible with asphalt than EUG overall since the surface of SEUG-modified asphalt has more folds, grooves, and SEUG particles of different sizes when given different amounts. This clearly demonstrates a non-homogeneous structure. The 5 wt% SEUG is only slightly cross-linked and mostly exists as small particles in the asphalt, as shown in Figure 23e, which shows a small number of folds and different-sized particles. In Figure 23f, 10 SA displays more uniform and smooth folds, and particulate matter is essentially undetectable. This shows that during the high-temperature and high-speed shear preparation process, the shear creates a lot of thermal and kinetic energy due to the quick friction between the rotor and the stator. This helps the SEUG break down into smaller and smaller pieces and encourages the SEUG to depolymerize [67]. In Figure 23g, numerous folds of varying lengths and widths are shown, along with irregularly shaped granular structures. This means that the SEUG depolymerization reaction has gone deeper, making more particles while absorbing more light components and making the structure even less uniform.

## 4. Conclusions

In this study, a combination of molecular simulations and laboratory experiments were used to investigate the effects of shear temperature, type of juniper gum, and amount of admixture on the compatibility of juniper gum with asphalt. Based on the experimental simulation results and discussions, the following conclusions can be drawn.

(1) The solubility parameters of gutta-percha and asphalt both decrease with temperature, and the solubility difference between EUG and asphalt is smaller than SEUG. At 165 °C, the solubility parameter difference between EUG and asphalt is at its lowest value, indicating that this is the temperature where EUG and asphalt are most compatible.
(2) As the temperature goes up, the electrostatic potential energy, non-bonding potential energy, and van der Waals potential energy of gutta-percha molecules all go up, then down, and then up again. The molecular potential energy fluctuation between EUG and asphalt is bigger than between SEUG, which means that the bonds between EUG and asphalt are stronger than between SEUG and asphalt.
(3) As the temperature rises, the diffusion coefficients *D* of EUG and SEUG in asphalt typically increase and then decrease. EUG moves through asphalt more quickly than SEUG because its *D* value is higher.
(4) The mechanical performance parameters of asphalt modified with 10 wt% SEUG were the best, and *E*, *K*, *G*, and *v* showed a tendency to rise and then fall with the amount of dulce de leche doped. It shows that even though SEUG and asphalt do not mix as well as EUG, SEUG-modified asphalt performs better mechanically.
(5) The three index tests, the segregation experiment, and the SEM test were also used to confirm the validity of the molecular dynamic simulation. Gutta-percha was found to significantly enhance the performance of asphalt at both high and low temperatures. When storing asphalt, EUG is less likely to segregate than SEUG, and 10 wt% EUG-modified asphalt has a smoother micromorphology.
(6) The mechanical, rheological, anti-fatigue, and anti-aging properties of gutta-percha-modified asphalt on a larger scale will be further investigated.

## Figures and Tables

**Table 1:** Technical information of eucommia ulmoides gum.

Technical Properties	Test Result	Specification Limits	Standards in Swiss
Density (g/cm^3^)	0.944	≥0.940	DIN 53479 [39]
Tensile strength (N/mm^2^)	38.20	≥25	DIN 53504 [40]
Elongation at break (%)	453.6%	≥400%	DIN 53504
Hardness (shore D)	46	≥40	DIN 53505 [41]
Modulus in tension (100%, N/mm^2^)	7.5	≥5	DIN 53504

**Table 2:** Technical properties of the base asphalt.

Technical Properties		Test Result	Specification Limits	Standard in China (JTG E20–2011) [43]
Penetration (25 °C, 0.1 mm)		90.5	80~100	T0604—2011
Softening point (R&B, °C)		47.7	≥40	T0606—2011
Ductility (5 °C, cm)		9.5	-	T0605—2011
Flashpoint (°C)		281	≥245	T0611—2011
Density (15 °C, g/cm^3^)		1.002	-	T0603—2011
Solution (Chloral, %)		99.25	≥99.5	T0607—2011
RTFOT Residuum	Mass loss rate (%)	0.05	≤±0.8	T0610—2011
	Penetration ratio (25 °C, %)	61.1	≥57	T0610—2011
	Ductility (5 °C, cm)	8.2	≥8	T0610—2011

**Table 3:** Molecular compositions of the AAA−1 asphalt model [47].

SARA	Molecules	Number in Model System	Molecular Representation	Molar Mass (g/mol)
Asphaltene	Phenol	3	C_42_H_54_O	575.0
	Pyrrole	2	C_66_H_81_N	888.5
	Thiophene	3	C_51_H_62_S	707.2
Aromatic	PHPN	11	C_35_H_44_	464.8
	DOCHN	13	C_30_H_46_	406.7
Resin	Quinolinohopane	4	C_40_H_59_N	553.9
	Thio-isorenieratane	4	C_40_H_60_S	573.0
	Benzobisbenzothiophene	15	C_18_H_10_S_2_	290.4
	Pyridinohopane	4	C_36_H_57_N	503.9
	Trimethylbenzene-oxane	5	C_29_H_50_N	414.7
Saturate	Squalane	4	C_30_H_62_	422.8
	Hopane	4	C_35_H_62_	482.9
Total number of atoms in the model		5572		
Lengths A, B, and C of lattice (Å)		37.82 × 37.82 × 37.82		

**Table 4:** Content and mass fraction of modifiers in the asphalt models.

Name	Asphalt Type	Quantity of Modifier	Modifier Mass Fraction (%)
BA	Base asphalt	0	0
5 EA	EUG-modified asphalt	1	5
10 EA		2	10
15 EA		3	15
5 SA	SEUG-modified asphalt	1	5
10 SA		2	10
15 SA		3	15

**Table 5:** The molecular potential energy of modifiers and bitumen models at different temperatures.

Molecular Species	Potential Energy (kJ/mol)	Temperature (°C)						
		105	120	135	150	165	180	195
EUG	*E_EP_*	127.65	291.85	406.67	448.02	572.41	671.70	747.86
	*E_EV_*	−418.31	−416.80	−382.26	−375.65	−370.45	−362.35	−332.11
	*E_Eε_*	−698.34	−698.61	−696.53	−697.16	−699.40	−699.33	−693.36
SEUG	*E_SP_*	1562.33	1762.20	1972.68	2096.02	2379.05	2538.66	2659.48
	*E_SV_*	−304.12	−298.91	−284.90	−276.63	−258.77	−213.81	−196.03
	*E_Sε_*	−1251.69	−1260.94	−1264.13	−1258.74	−1267.07	−1261.42	−1254.38
BA	*E_AP_*	10,594.24	11,112.35	11,243.73	11,628.45	11,828.59	12,074.78	12,362.09
	*E_AV_*	−806.12	−698.18	−720.82	−681.41	−641.24	−567.73	−571.73
	*E_Aε_*	−861.26	−863.80	−873.98	−862.87	−863.76	−868.76	−862.02
5 EA	*E_5EAP_*	11,000.09	11,700.01	11,979.95	12,405.51	12,801.67	13,060.26	13,403.95
	*E_5EA V_*	−957.88	−861.55	−772.69	−707.42	−600.02	−577.61	−554.05
	*E_5EAε_*	−1268.95	−1266.79	−1270.43	−1268.40	−1208.68	−1237.87	−1261.52
10 EA	*E_10EAP_*	11,067.45	11,718.57	11,890.74	12,367.08	12,845.84	13,056.05	13,418.74
	*E_10EAV_*	−833.80	−755.84	−709.15	−663.79	−559.45	−550.54	−532.62
	*E_10EAε_*	−1255.59	−1232.89	−1247.46	−1239.58	−1182.90	−1239.83	−1231.83
15 EA	*E_15EAP_*	11,025.63	11,724.47	11,968.83	12,401.72	12,776.36	13,075.37	13,384.18
	*E_15EAV_*	−857.62	−814.36	−753.75	−733.25	−604.41	−683.88	−551.77
	*E_15EAε_*	−1294.72	−1291.10	−1292.18	−1288.87	−1227.51	−1275.36	−1284.93
5 SEA	*E_5SAP_*	12,309.123	13,040.353	13,385.187	13,875.004	14,368.156	14,793.358	15,175.237
	*E_5SAV_*	−925.33	−832.45	−822.45	−760.25	−702.73	−556.86	−545.40
	*E_5SAε_*	−2008.26	−2003.842	−2002.518	−1992.508	−1996.775	−1990.716	−1992.89
10 SEA	*E_10SAP_*	12,314.337	13,015.27	13,347.50	13,868.18	14,362.52	14,782.266	15,162.63
	*E_10SAV_*	−883.677	−810.994	−788.414	−745.369	−680.3	−525.546	−514.832
	*E_10SAε_*	−1975.95	−1986.67	−2004.05	−1989.41	−2002.88	−1981.52	−1984.76
15 SA	*E_15SAP_*	12,309.41	13,020.69	13,385.76	13,900.25	14,361.05	14,795.38	15,187.27
	*E_15SAV_*	−905.75	−791.04	−780.28	−721.955	−675.083	−540.62	−529.789
	*E_15SAε_*	−1994.48	−1999.299	−1992.8	−1996.481	−1983.736	−1980.613	−1980.083

**Table 6:** Mechanical properties of different asphalt species.

Asphalt Species	λ	μ	*E* (GPa)	*K* (GPa)	*G* (GPa)	*ν*
BA	2.1042	1.2713	3.3351	2.6286	1.2713	0.3117
5 EUG AS	2.2422	1.2878	3.3936	2.7599	1.2878	0.3176
10 EUG AS	2.8347	1.3520	3.6194	3.3278	1.3520	0.3385
15 EUG AS	2.6788	1.2988	3.4723	3.1921	1.2988	0.3367
5 SEUG AS	2.7477	1.4301	3.8008	3.2139	1.4301	0.3288
10 SEUG AS	2.8109	1.6289	4.2891	3.2202	1.6289	0.3166
15 SEUG AS	2.7890	1.4840	3.9366	3.2382	1.4840	0.3264

## Data Availability

Not applicable.
